# Do positions in individual-based ectoparasite-small mammal networks differ between female and male hosts?

**DOI:** 10.1007/s00436-025-08492-2

**Published:** 2025-04-29

**Authors:** Boris R. Krasnov, Maxim V. Vinarski, Natalia P. Korallo-Vinarskaya, Michal Stanko

**Affiliations:** 1 Mitrani Department of Desert Ecology, Swiss Institute for Dryland Environmental and Energy Research, Jacob Blaustein Institutes for Desert Research, Ben-Gurion University of the Negev, Sede Boqer Campus, 84990 Beer-Sheva, Midreshet Ben-Gurion Israel; 2 Laboratory of Macroecology and Biogeography of Invertebrates, Saint-Petersburg State University, Saint-Petersburg, Russian Federation; 3 Laboratory of Parasitology, Zoological Institute of the Russian Academy of Sciences, Saint-Petersburg, Russian Federation; 4 Institute of Parasitology, Slovak Academy of Sciences, Kosice, Slovakia; 5 Institute of Zoology, Slovak Academy of Sciences, Bratislava, Slovakia

**Keywords:** Host-parasite network, Fleas, Gamasid mites, Small mammals, Structure

## Abstract

We investigated the contributions of female and male hosts to the structure of individual-based host-parasite networks, using 21 species of small mammals from two regions (West Siberia and eastern Slovakia) and two taxa of ectoparasitic arthropods (fleas and gamasid mites). We asked whether (a) the values of individual host position indices (individual strength, nested rank, the degree of individual specialization, and the eigenvector centrality) and individual host roles differed between female and male hosts in each network and (if yes) were associated with differences in the infestation levels, (b) differences between sexes (if any) were further translated into differences in the network structure (nestedness and network specificity), and (c) differences between female and male hosts in their positions and roles and the effect of these differences on the network structure differed between host-flea and host-mite networks. In the majority of individual-based host-ectoparasite networks, female and male hosts differed in their positions despite a general lack of differences in the infestation levels. The distribution of the roles played in a network mostly did not differ between sexes. The extent of position differences between sexes affected the structure of host-flea, but not host-mite, networks in terms of the network specificity. In addition, the occurrence and the direction of these differences (i.e., the greater value of the position index in either female or male hosts) often varied (a) between host-flea and host-mite networks in the same host, (b) between host species within host-flea and host-mite networks, and (c) between the two regions in the same host-flea or host-mite network. We conclude that differences in the positions of male and female hosts in individual-based host-parasite networks are mediated by host biology, parasite biology, and environmental factors.

**Supplementary Information:**

The online version contains supplementary material available at 10.1007/s00436-025-08492-2.

## Introduction

Sex-biased parasitism is one of the most common parasitological phenomena (Zuk and McKean 1996; Poulin 1996a, b; Perkins et al. 2008; Krasnov et al. 2012; Cozzarolo et al. 2019; Rosso et al. 2020). The term “sex-biased” parasitism is often synonymised with “male-biased” parasitism (e.g., Matthee et al. 2009; Stephenson et al. 2016). This is because, in many host-parasite associations, especially small mammals, males are more heavily parasitized than females in terms of parasite abundance, prevalence, and/or species richness (Behnke et al. 1999; Moore and Wilson 2002; Morand et al. 2004; Krasnov et al. 2005). The proposed mechanisms for this are (a) the greater androgen levels in males, which suppress the immune function (the so-called immunological handicap hypothesis), thus making males more vulnerable to parasites (Folstad and Karter 1992; Zuk 1996; Zuk and McKean 1996), (b) the greater mobility of males (particularly, in mammals), which increases their chances of encountering parasites (Krasnov et al. 2005), and (c) the larger male body size in many species, causing males to be larger targets for parasites and to trade off energy expenditure for growth and immunity (Morand et al. 2004; Kiffner et al. 2011). These mechanisms likely act together rather than separately (Krasnov et al. 2012). However, male-biased parasitism, albeit frequently observed, is not universal. In fact, in bat-batfly and bat-mite associations, females are often more heavily parasitized than males (Dick et al. 2003; Christe et al. 2007). Moreover, manifestations of male- or female-biased parasitism can vary between parasite taxa in the same host species (Hillegass et al. 2008; Matthee et al. 2009) or in the same host-parasite association, depending on environmental factors (Krasnov and Matthee 2010).

Sex-biased parasitism has important consequences for host and parasite individuals (Christe et al. 2007; Córdoba-Aguilar and Munguía-Steyer 2013), populations (Ferrari et al. 2004), and communities (Krasnov et al. 2011). For example, Krasnov et al. (2011) reported that the non-randomness of Palearctic rodents’ flea infracommunities was expressed more strongly in male than in female hosts, suggesting that males were the main drivers of this structural pattern. Sex-biased parasitism has been reported to be accompanied by differential roles played by male and female hosts in parasite transmission (e.g., Grear et al. 2009, 2012). This suggests that male and female hosts may take different positions and/or play different roles in individual-based host-parasite networks (Godfray 2013; Xu et al. 2022). As a result, male and female hosts may not be equally important in the processes acting in the networks (Delmas et al. 2019). Consequently, differences in the positions of male and female hosts in individual-based networks may affect the network’s structure. To the best of our knowledge, this has never been studied in host-parasite networks.

Here, we attempted to fill this gap by investigating the contributions of female and male hosts to the structure of individual-based host-parasite networks, using 21 species of small mammals from two regions (West Siberia and eastern Slovakia) and two taxa of ectoparasitic arthropods (fleas and gamasid mites) as model associations. We characterized individual host positions using four indices (individual strength, nested rank, the degree of individual-level interaction specialization, and the eigenvector centrality; see definitions in the “Materials and Methods” section). Bipartite ecological networks (such as host-parasite networks) are characterized by a greater or lesser degree of modularity (i.e., the degree of network subdivision into separate subsets of frequently interacting species) (Bascompte and Jordano 2013). Host individual roles within a network were classified based on the interactions of each host individual with parasite species within its module and its interactions with parasites from other modules (Guimerà and Amaral 2005). We described the structure of each network using two indices (nestedness and network specificity). A nested network consists of a core group of generalist species interacting with each other and with strict specialist species that only interact with generalists (Nielsen and Bascompte 2007). The degree of specificity of a host-parasite network depends on and highly correlates with the level of parasite host specificity (Blüthgen et al. 2006). We asked whether (a) the values of individual host position indices and individual host roles differed between female and male hosts in each network and (if yes) were associated with differences in the infestation levels, (b) differences in the network positions between sexes (if any) were further translated into differences in the network structure, and (c) differences between female and male hosts in their positions and roles and the effect of these differences on the network structure differed between host-flea and host-mite networks.

## Materials and methods

### Data on flea and gamasid mite distributions across host individuals

Data on flea and gamasid mite distributions across individual hosts were taken from surveys of ectoparasites of rodents and shrews carried out in 1960–2013 in West Siberia (compiled from the database of the Omsk Research Institute of Natural Foci Infections (Omsk, Russian Federation) by NPKV) and in 1986–2000 in eastern Slovakia. For mites, we took into account only obligatory and facultatively haematophagous species collected from host bodies. Details on sampling design and methods of ectoparasite collection and identification can be found in Krasnov et al. (2019) for Siberia and Stanko et al. (2002) for Slovakia. In Siberia and Slovakia, sampling was carried out across 108 and 13 sampling sites, respectively. For further analyses, we selected host species in which at least 20 male and 20 female individuals, from the same sampling site across all sampling periods, were found to be parasitized by at least one flea or one gamasid mite species. This resulted in the selection of 15 host species from Siberia and six host species from Slovakia (Tables S1–S2 in the Electronic Supplementary Material). Siberian host species were *Apodemus agrarius*, *Arvicola amphibius*, *Craseomys rufocanus*, *Microtus agrestis*, *Microtus arvalis*, *Microtus gregalis*, *Microtus middendorffii*, *Microtus oeconomus*, *Myodes glareolus*, *Myodes rutilus*, *Sicista betulina*, *Sorex araneus*, *Sorex isodon*, *Sorex minutus*, and *Sorex tundrensis*. Slovakian species were *A. agrarius*, *Apodemus flavicollis*, *Apodemus uralensis*, *M. arvalis*, *M. glareolus*, and *S. araneus*. These hosts were parasitized by 29 flea and 38 mite species in Siberia and by 26 flea and 17 gamasid mite species in Slovakia (Tables S3–S4 in the Electronic Supplementary Material).

### Individual host infestation levels and positions in networks

For each host and either flea or mite species, we constructed an interaction network, i.e., a matrix of interactions, with parasite species in columns and host individuals in rows and with entries being the numbers of individual parasites of a given species recorded on a given individual host. For host species that occurred in both Siberia and Slovakia, this was done separately for each region. For each host individual in each network, we calculated the numbers of flea or mite individuals and species. Then, we calculated an individual host position in a network using four indices, namely species strength, nested rank, the degree of species-level interaction specialization *d′*, and the eigenvector centrality. Species strength (= individual host strength, in our case) (Jordano 1987; Bascompte et al. 2006) is a quantitative measure of a given resource’s relevance (i.e., an individual host) for a consumer community (all parasite species). It is calculated as the sum of the dependencies (the relative frequency of interactions) of all parasite species on that individual host. A host individual’s nested rank is the rank of this individual in a network matrix re-arranged for maximal nestedness (Alarcon et al. 2008). In our case, a perfectly (i.e., maximally) nested interaction matrix is a matrix in which specialist individual hosts interact with parasite species that represent perfect subsets of parasites with which generalist individual hosts interact. The degree of species-level (individual-level, in our case) specialization (*d′*) (further referred to as the degree of specialization) (Blüthgen et al. 2006, 2007; Blüthgen 2010) compares the frequency distribution of an individual host’s interactions with a null distribution when this host interacts with the random parasite sets, thus describing the deviation from a neutral configuration of associations. All the abovementioned indices were calculated using the “specieslevel” function of the “bipartite” (ver. 2.21) package (Dormann 2011; Dormann et al. 2008) with the option level = “lower” (i.e., for hosts and not for parasites) and nested.normalise = TRUE. The latter option divides the nested rank − 1 by the number of species − 1, so that the nested rank ranges between 0 (most generalist) and 1 (most specialized). The eigenvector centrality is a measure of a species’ influence in a network that takes into account the importance of its neighbors (Bonacich 1987; Jordán and Scheuring 2004; Allesina and Pascual 2009; Delmas et al. 2019). Each network (bipartite, by definition) was projected to a unipartite network and transformed into the network graph object, using the “projecting_tm” and “tnet_igraph” functions, respectively, of the R package “tnet” (ver. 3.0.16) (Opsahl 2009). Then, the eigenvector centrality was calculated using the “evcent” function of the package “igraph” (ver.1.5.1) (Csardi and Nepusz 2006; Csárdi et al. 2023) implemented in the R (ver. 4.4.2) statisticalenvironment (R Core Team 2024).

### Individual host roles in networks

To define the roles of each individual host (sensu Stouffer et al. 2012), we computed modules for each network (groups of nodes that are more connected to each other than to other nodes), using the “computeModules” function of the “bipartite” package. Then, the role of an individual host was identified according to its location in a two-dimensional space of within-module degree *z* and between-module connectivity *c* (= participation coefficient *P* of Guimerà and Amaral (2005)). Species characterized by (a) low *z* and *c* were considered as peripherals, (b) low *z* and high *c* as connectors, (c) high *z* and low *c* as module hubs, and (d) high *z* and high *c* as network hubs. Critical values of *z* and *c* were calculated based on the distribution of the data as 95 percentiles, separately for each network. Then, *z* and *c* values for each host individual were calculated using the “czvalues” function of the “bipartite” package.

### Network structures

We characterized the structure of each network using two indices, namely nestedness and network specificity (*H2′*). Nestedness was calculated as the NODF index (nestedness based on paired overlap and decreasing fill; Almeida-Neto et al. 2008). Network specificity (*H2′*) is a network-level degree of specialization ranging between zero and unity (complete specialization), calculated in comparison to no specializations (Blüthgen et al. 2006). These indices were computed with the “networklevel” function of the “bipartite” package. Because networks differed in the numbers of host individuals, parasite species, and interactions, we standardized the indices using null models (with the “vaznull” function of the “bipartite” package, constructing 1000 reshuffled matrices) and *Z*-scores.

### Data analyses

To test for the within-network difference between female and male hosts in the numbers of ectoparasite individuals, we applied negative binomial regression using the R package “MASS” (ver. 7.3–65) (Venables and Ripley 2002). The difference between sexes in the number of parasite species was tested with generalized linear models using the R package “stats” with the option “family = poisson.” Then, we tested for the differences between male and female hosts in their network positions (separately for each host-flea and host-mite network), represented by a combination of the four abovementioned indices, applying distance-based multivariate analyses of variance (db_MANOVAs), implemented in the R package “PERMANOVA” (ver. 0.2.0) (Vicente-Gonzalez and Vicente-Villardon 2021). Db_MANOVA is a non-parametric multivariate generalization of a traditional ANOVA that allows comparing the within-group and the between-group dissimilarities (Anderson 2001) and, thus, testing for the differences between two or more groups. Pairwise dissimilarities, in the combination of the position indices between female and male hosts of the same species, were calculated using the Pythagorean coefficient, and the *p*-values were obtained by 10,000 random permutations. However, db-MANOVA did not provide information on which specific position index contributes the most to female-male differences (if any) in their network positions. Therefore, we further tested for significant differences between female and male positions, described by each of the position indices, by applying univariate permutational ANOVAs with 10,000 random permutations of host individuals between sexes. This was carried out using the R package “RRPP” (ver. 2.1.2) (Collyer and Adams 2018).

For the across-network analyses, we calculated the proportions of individual female or male hosts playing one of the four network roles for each network. Then, we compared the proportions of individual hosts playing a given role between females and males across all host species, using generalized linear mixed models (GLMM), with species as a random factor and the beta distribution implemented in the R package “glmmTMB” (ver. 1.11.11) (Brooks et al. 2017). Because the beta distribution considers the distribution of the variable ranging from 0 to 1 and not attaining these extreme values, prior to analyses, we added 0.0000001 to all 0 s and subtracted 0.0000001 from all 1 s.

We tested whether network structure, represented by nestedness and network specificity, is affected by differences between female and male positions. We calculated sex differences in each position index, in each network, as the natural logarithm of the mean male-to-female or female-to-male ratio (depending on whether the mean value of an index was greater in males or females). Because our data represented values for different species, we applied phylogenetic generalized linear models (PGLS; Freckleton et al. 2002), with NODF or H2′ as a response variable and the four position indices as explanatory variables. This was done separately for host-flea and host-mite networks. A host phylogenetic tree (topologies and branch lengths) was constructed from a subset of 1000 trees, taken randomly from Upham et al.’s (2019) 10,000 species-level birth–death tip-dated completed trees for 5911 mammal species (https://vertlife.org/). From this subset, a consensus tree was built using the “consensus.edge” function of the R package “phytools” (ver. 2.4–4) (Revell 2012). The host species that occurred in both Siberia and Slovakia were considered sister species, and thus, a length of 1 was assigned to all branches. PGLSs were run using the R package “mmodely” (ver. 0.2.5) (Schruth 2023). First, we compiled a list of models with all possible combinations of the explanatory variables, using the “get.model.combos” function. Then, we ran all these models with the “pgls.iter” function and selected the best model based on Akaike information criterion, using the “select.best.models” function. Finally, we ran the best model separately for each of the two indices of the network structure and for host-flea and host-mite networks.

We intentionally did not apply the adjustment of the alpha level for multiple comparisons (e.g., Bonferroni corrections). Although this can easily be done, this procedure has been strongly criticized by both statisticians and ecologists because it can inflate the rate of type II errors (Rothman 1990; Perneger 1998; Moran 2003; Garcia 2004; Nakagawa 2004).

## Results

Median values with interquartile ranges of the numbers of flea and mite individuals and species collected from each individual female or male host are presented in Supplementary Tables S5–S8. Significant differences between female and male hosts in counts of ectoparasite individuals were detected in a few host species only. For fleas, they were Siberian *M. agrestis* and *M. gregalis* and Slovakian *A. flavicollis* (*z* = 3.11–4.62, respectively, *p* < 0.001 for all). For mites, significant difference between sexes were found in three Siberian hosts (*A. agrarius*, *M. gregalis*, and *M. rutilus*; *z* = − 2.15–7.69, *p* < 0.03 for all) and three Slovakian hosts (*A. uralensus*, *M. arvalis*, and *M. glareolus*; *z* = − 4.95–7.68, *p* < 0.00q for all). Whenever the difference between female and male hosts was significant, the counts of fleas were greater in males than in female hosts, whereas the counts of mites were greater in males of *M. gregalis*, *M. arvalis*, and *M. glareolus*, but in females of the remaining three host species. Significant differences in ectoparasite species richness were detected in one Siberian and one Slovakian host for fleas (*M. gregalis* and *A. flavicollis*, respectively; *z* = 1.99–2.16, *p* < 0.05 for both) and one Siberian host for mites (*M. gregalis*; *z* = 2.61,* p* = 0.01). In all three species, male hosts harbored a greater number of ectoparasite species.

The results of the db_MANOVAs demonstrated that individual host positions, measured as a combination of the four position indices, differed significantly between female and male hosts (a) in nine of 15 Siberian hosts and three of six Slovakian hosts in host-flea networks (Table 1) and (b) in seven of 15 Siberian hosts and five of six Slovakian hosts (Table 2) in host-mite networks. In the same host species occurring in both regions, a difference between females and males in their network position was detected either in both regions (e.g., *A. agrarius* in host-flea networks; Table 1) or in one of the two regions only (e.g., *M. arvalis* in host-mite networks; Table 2).

**Table 1:** Summary of distance-based multivariate analyses of variance (db-MANOVAs) testing for differences between male and female hosts in their network properties (degree of species specialization, nested rank, individual strength, and the eigenvector centrality) in the individual-based host-flea networks from two regions

Region	Species	Pseudo-*F*	*p*
Siberia	*A. agrarius*	16.09	< 0.001
	*A. amphibius*	0.45	0.64
	*C. rufocanus*	1.18	0.29
	*M. agrestis*	3.78	0.02
	*M. arvalis*	0.50	0.60
	*M. gregalis*	3.36	0.04
	*M. middendorffii*	3.32	0.03
	*M. oeconomus*	1.12	0.31
	*M. glareolus*	0.66	0.53
	*M. rutilus*	12.82	< 0.001
	*S. betulina*	4.95	0.01
	*S. araneus*	11.12	< 0.001
	*S. isodon*	3.25	0.03
	*S. minutus*	3.61	0.04
	*S. tundrensis*	0.65	0.55
Slovakia	*A. agrarius*	20.07	< 0.001
	*A. flavicollis*	0.24	0.82
	*A. uralensis*	4.51	0.02
	*M. arvalis*	3.76	0.03
	*M. glareolus*	1.05	0.30
	*S. araneus*	0.80	0.44

**Table 2:** Summary of distance-based multivariate analyses of variance (db-MANOVAs) testing for differences between male and female hosts in their network properties (degree of species specialization, nested rank, individual strength, and the eigenvector centrality) in the individual-based host-mite networks from two regions

Region	Species	Pseudo-*F*	*p*
Siberia	*A. agrarius*	2.89	0.04
	*A. amphibius*	0.56	0.58
	*C. rufocanus*	1.19	0.32
	*M. agrestis*	0.89	0.40
	*M. arvalis*	1.14	0.35
	*M. gregalis*	8.44	< 0.001
	*M. middendorffii*	0.55	0.58
	*M. oeconomus*	1.60	0.15
	*M. glareolus*	3.63	0.04
	*M. rutilus*	12.51	< 0.001
	*S. betulina*	2.90	0.04
	*S. araneus*	2.90	0.04
	*S. isodon*	4.03	0.02
	*S. minutus*	0.37	0.72
	*S. tundrensis*	0.66	0.51
Slovakia	*A. agrarius*	7.24	< 0.001
	*A. flavicollis*	3.44	0.04
	*A. uralensis*	6.78	< 0.001
	*M. arvalis*	3.62	0.04
	*M. glareolus*	2.72	0.06
	*S. araneus*	3.33	0.03

**Fig. 1:**
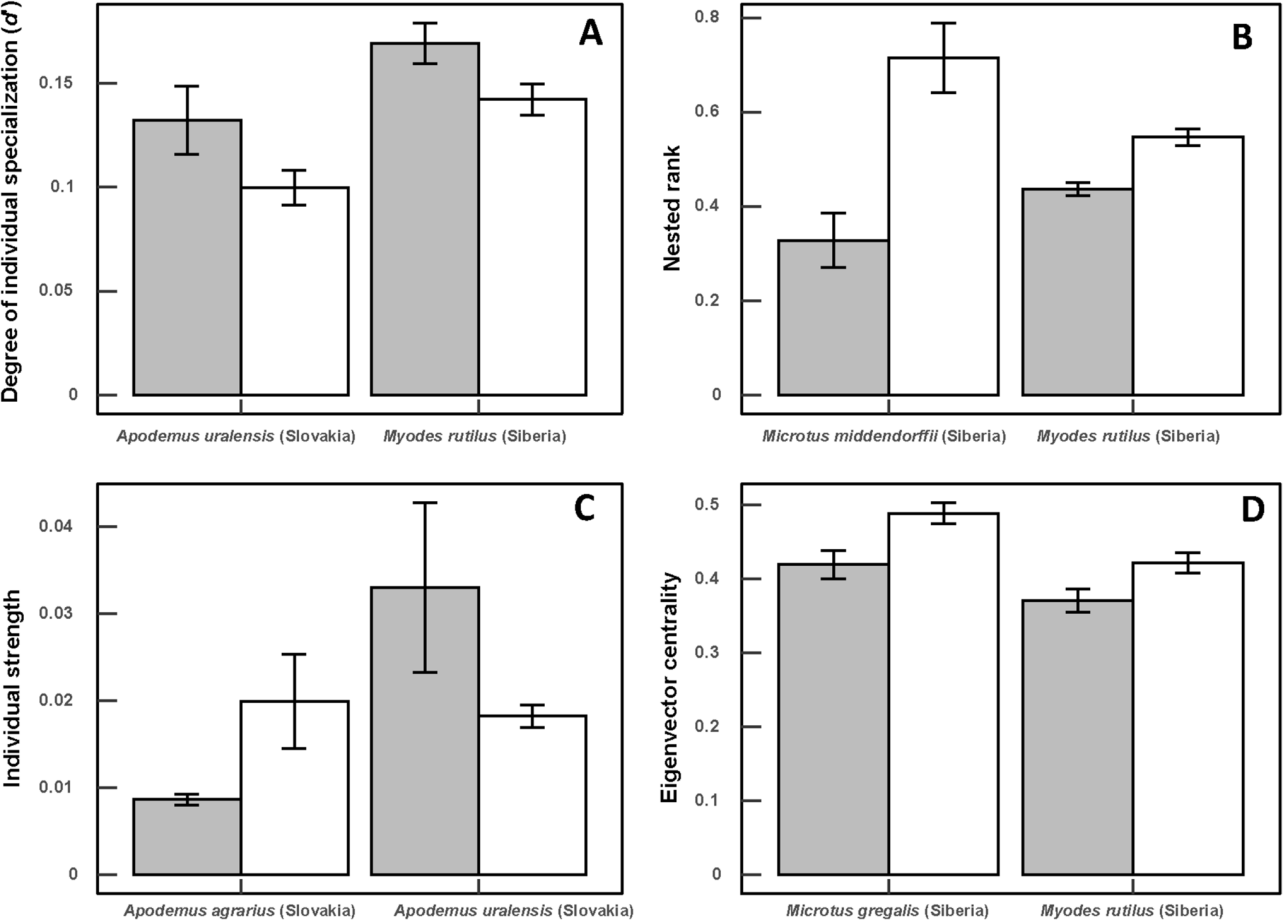
Illustrative examples demonstrating mean (± S.E.) values of **A** the degree of specialization *d′*; **B** nested rank; **C** individual strength; and **D** the eigenvector centrality of female (white bars) and male (gray bars) host individuals in host-flea networks from two regions

In all hosts for which a significant difference between sexes in combined position indices was detected, these differences between females and males were found in one to all four separate indices (Tables 3, 4). In host-flea networks, values of the degree of specialization differed significantly between females and males in two species (being greater in male hosts; see illustrative example in Fig. 1A), values of nested rank in 10 species (greater in female hosts; Fig. 1B), values of individual strength in two species (greater in either female or male hosts; Fig. 1C), and values of eigenvector centrality in four species (greater in male hosts, Fig. 1D) (Table 3).

**Table 3:** Mean (± S.E.) values of the network position indices of female (Fl) and male (Ml) hosts in the individual-based host-flea networks from two regions. The results are shown only for hosts and indices for which significant differences between males and females were detected. Indices are as follows: *NR*, nested rank; *d′*, the degree of specialization; *IS*, individual strength; *EC*, the eigenvector centrality

Region	Species	Index	Fl	Ml	*F*	*p*
Siberia	*A. agrarius*	NR	0.43 ± 0.03	0.54 ± 0.03	6.06	0.02
	*M. agrestis*	EC	0.40 ± 0.03	0.51 ± 0.03	5.70	0.02
	*M. gregalis*	EC	0.42 ± 0.02	0.51 ± 0.01	7.94	0.004
	*M. middendorffii*	NR	0.33 ± 0.06	0.71 ± 0.07	17.45	< 0.001
	*M. rutilus*	*d′*	0.17 ± 0.01	0.14 ± 0.01	4.99	0.02
		NR	0.44 ± 0.01	0.55 ± 0.02	20.45	< 0.001
		EC	0.32 ± 0.02	0.42 ± 0.01	5.82	0.01
	*S. betulina*	NR	0.36 ± 0.03	0.55 ± 0.03	12.16	< 0.001
	*S. araneus*	NR	0.40 ± 0.02	0.58 ± 0.03	23.26	< 0.001
	*S. isodon*	NR	0.44 ± 0.04	0.56 ± 0.04	4.95	< 0.001
	*S. minutus*	NR	0.40 ± 0.03	0.58 ± 0.04	9.33	< 0.001
Slovakia	*A. agrarius*	NR	0.43 ± 0.01	0.55 ± 0.02	24.70	< 0.001
		IS	0.01 ± 0.0006	0.02 ± 0.01	3.35	0.04
	*A. uralensis*	*d′*	0.13 ± 0.02	0.10 ± 0.0008	3.84	0.04
		NR	0.44 ± 0.02	0.53 ± 0.03	4.63	0.04
		IS	0.03 ± 0.01	0.018 ± 0.001	4.12	0.03
		EC	0.39 ± 0.03	0.46 ± 0.02	4.49	0.04
	*M. arvalis*	NR	0.47 ± 0.02	0.54 ± 0.03	5.23	0.02

**Table 4:** Mean (± S.E.) values of the network position indices of female (Fl) and male (Ml) hosts in the individual-based host-mite networks from two regions. The results are shown only for hosts and indices for which significant differences between males and females were detected. Indices are as follows: *NR*, nested rank; *d′*, the degree of specialization; *IS*, individual strength; *EC*, the eigenvector centrality

Region	Species	Index	Fl	Ml	*F*	*p*
Siberia	*A. agrarius*	NR	0.44 ± 0.03	0.54 ± 0.03	4.88	0.02
	*M. arvalis*	*d′*	0.23 ± 0.03	0.33 ± 0.03	3.74	0.04
	*M. gregalis*	NR	0.55 ± 0.02	0.47 ± 0.02	8.58	< 0.001
		EC	0.45 ± 0.02	0.52 ± 0.01	14.54	< 0.001
	*M. oeconomus*	NR	0.44 ± 0.03	0.55 ± 0.04	3.50	0.02
	*M. glareolus*	NR	0.41 ± 0.03	0.59 ± 0.05	8.22	< 0.001
	*M. rutilus*	NR	0.39 ± 0.02	0.57 ± 0.02	34.58	< 0.001
	*S. betulina*	NR	0.30 ± 0.03	0.55 ± 0.03	12.20	< 0.001
	*S. araneus*	NR	0.46 ± 0.02	0.54 ± 0.03	4.94	0.02
	*S. isodon*	*d′*	0.30 ± 0.06	0.13 ± 0.04	5.13	0.03
		EC	0.49 ± 0.09	0.76 ± 0.07	6.10	0.01
Slovakia	*A. agrarius*	NR	0.46 ± 0.01	0.53 ± 0.02	13.01	< 0.001
	*A. flavicollis*	*d′*	0.21 ± 0.02	0.15 ± 0.01	8.38	< 0.001
		NR	0.44 ± 0.02	0.54 ± 0.02	8.65	< 0.001
		IS	0.06 ± 0.01	0.03 ± 0.0002	10.11	< 0.001
		EC	0.60 ± 0.01	0.65 ± 0.01	7.22	0.01
	*M. arvalis*	*d′*	0.17 ± 0.01	0.14 ± 0.01	6.91	0.01
		EC	0.41 ± 0.01	0.47 ± 0.01	8.18	0.004
	*M. glareolus*	EC	0.36 ± 0.03	0.44 ± 0.02	5.80	0.02
	*S. araneus*	NR	0.27 ± 0.03	0.62 ± 0.06	18.96	< 0.001

**Fig. 2:**
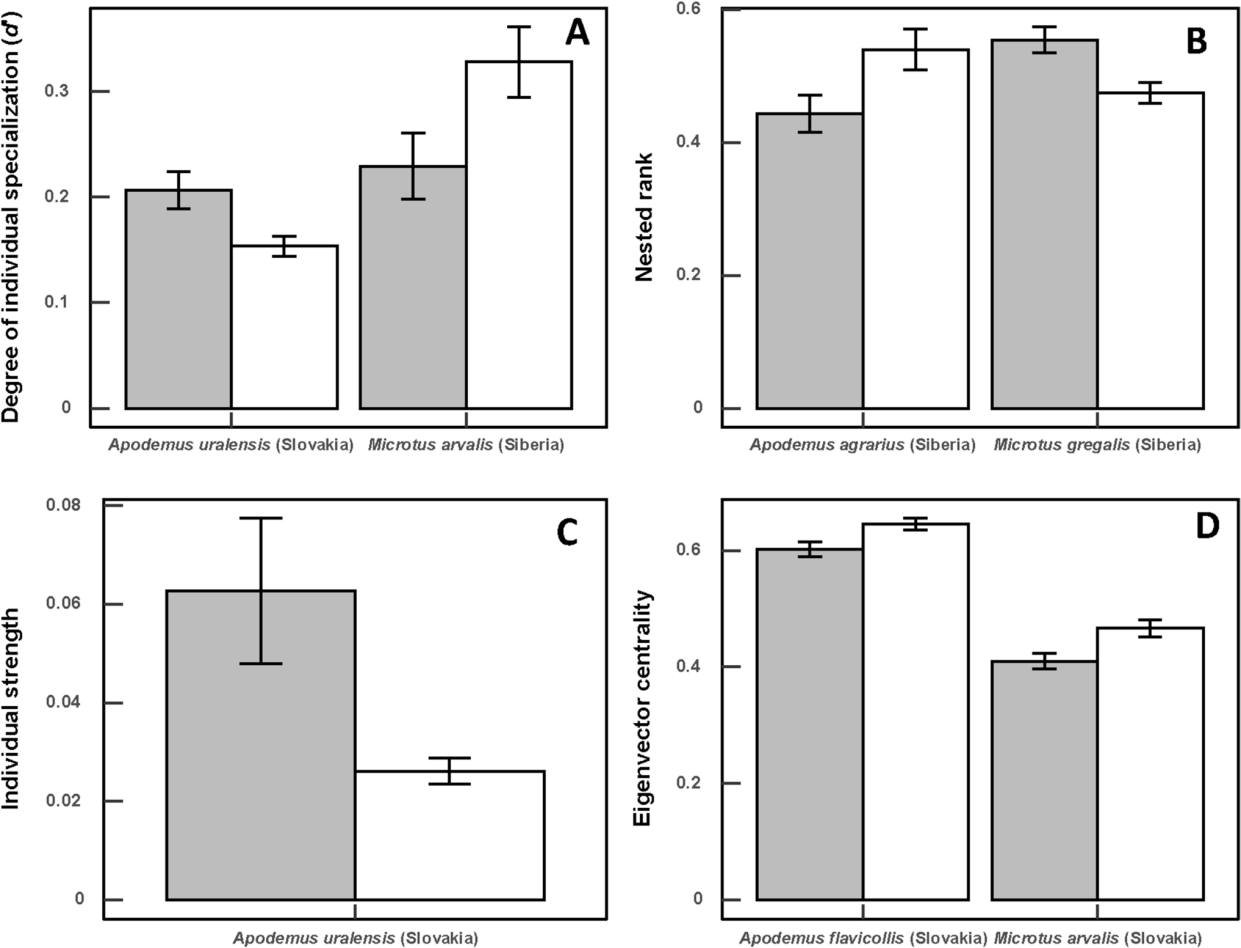
Illustrative examples demonstrating mean (± S.E.) values of **A** the degree of specialization *d′*; **B** nested rank; **C** individual strength; and **D** the eigenvector centrality of female (gray bars) and male (white bars) host individuals in host-mite networks from two regions

In host-mite networks, values of the degree of specialization differed significantly between sexes in four species (greater in male hosts in three of them; see illustrative example in Fig. 2A), values of nested rank in 10 species (greater in male hosts in nine of them; Fig. 2B), values of individual strength in one species (Slovakian *A. uralensis*, greater in female hosts; Fig. 2C), and values of eigenvector centrality in five species (Fig. 2D) (Table 4).

**Fig. 3:**
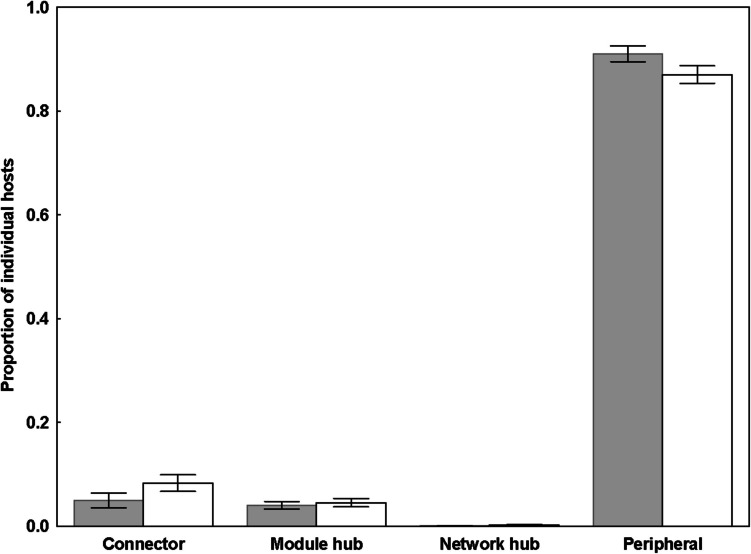
Proportions of female (gray bars) and male (white bars) host individuals playing different roles in host-mite networks

The proportion of individual hosts playing different roles did not differ between females and males in host-flea networks (GLMM, *z* = 0.38, *p* = 0.70). On the contrary, these proportions differed significantly between sexes in host-mite networks (GLMM, *z* = 3.20, *p* = 0.001). Proportions of the connector and peripheral males were greater and lesser (albeit not substantially), respectively, than those of the connector and peripheral females (Fig. 3).

**Fig. 4:**
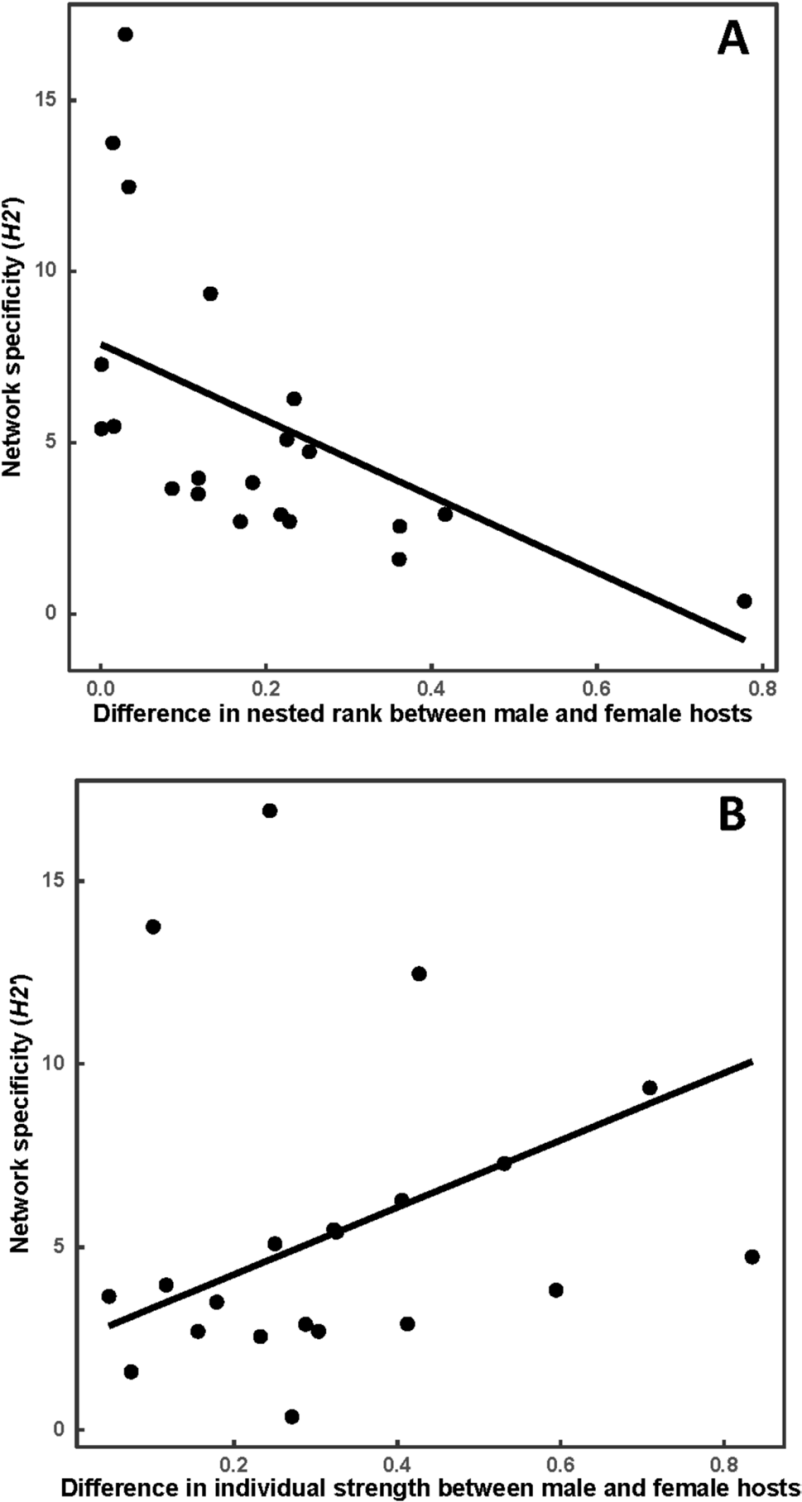
Relationships (partial regression fits; see Table 5) between the specificity of individual-based host-flea networks (*H2′*) and the differences between female and male individuals in nested rank (**A**) and individual strength (**B**)

The best models explaining the effects of the differences between female and male hosts in their position index values on the network structure are presented in Table 5. The degree of nestedness in host-flea networks was not affected by differences between female and male hosts in any position index. However, larger differences between the sexes in nested rank and individual strength corresponded to lesser and greater degrees of network specificity, respectively (Fig. 4). In host-mite networks, no effect of female-male differences in their positions was detected for either nestedness or network specificity.

**Table 5:** The best models of the effects of female-male differences in the values of position indices on the indices of the structure of individual-based host-flea and host-mite networks. *NODF*, the degree of nestedness; *H2′*, network specificity; *dEC*, difference in the eigenvector centrality; *dSS*, difference between sexes in mean individual strength; *dNR*, difference between sexes in mean nested rank. Coefficients and their statistics are only shown for significant predictors; *p*_c_,* p*-value for a coefficient; *p*_m_,* p*-value for the whole model

Parasite	Index of structure	Equation	*t*	*p*_c_	*R*^*2*^	*F*	*p*_m_
Fleas	NODF	dEC + dSS			0.28	3.10	0.07
	*H2′*	− 9.35*dNR + 7.37*dSS	− 3.07, 2.64	0.01, 0.02	0.56	10.22	0.01
Mites	NODF	dSS			0.12	2.14	0.13
	*H2′*	dSS			0.09	1.65	0.21

## Discussion

We found that in the majority of individual-based host-ectoparasite networks, female and male hosts differed in their positions despite a general lack of differences in infestation levels, although the distribution of the roles played in a network mostly did not differ between the sexes. The extent of position differences between the sexes affected the structure of host-flea, but not host-mite, networks, in terms of the network specificity. In addition, the occurrence and the direction (i.e., the greater value of the position index in either female or male hosts) often differed (a) in the same host between host-flea and host-mite networks, (b) between host species within host-flea and host-mite networks, and (c) in the same host’s host-flea or host-mite networks between the two regions.

Among the indices of individual host position in a network, differences between sexes were most often found in the network rank of an individual host, with this rank being, in general, greater in males. Given the definition and the method of calculating this index (see above), this demonstrated that male hosts were more specialized in terms of interactions with the parasites than female hosts. In many hosts, these differences were not revealed when the mere numbers of ectoparasite individuals and species were compared between sexes. This is because the nested rank of each individual host considers not only the number of its links with parasites but also depends on the interactions by all other individuals in a network. The greater nested rank of male hosts suggests that they tended to be exploited by a subset of parasites exploiting female hosts (Bascompte et al. 2003). This could be because some parasite species likely prefer to exploit female rather than male hosts, whereas other parasites do not demonstrate any preference for host sex. For example, in experiments with the mite *Spinturnix andegavinus* and the bat *Myotis daubentoni*, mites preferentially selected female hosts (Christe et al. 2007). In contrast, the selection of male over female hosts has been demonstrated in experiments for the flea *Xenopsylla ramesis* and the gerbil *Meriones crassus* (Khokhlova et al. 2011). However, this discrimination was characteristic only for female and not male fleas.

In five of the six host-ectoparasite networks in which a significant difference in the degree of specialization between female and male hosts was detected, the mean value of this index was greater in females. Although the greater nested rank of male hosts demonstrates that they are more specialized than females in terms of their role in the nestedness pattern, females appeared to be more specialized in terms of the diversity of partners and their availability, as indicated by the degree of specialization values (Blüthgen et al. 2006). This contradiction suggests that nested rank and *d′* capture different facets of specialization (Dormann 2011). In the same host-parasite network, males could be more specialized than females in the network context (nested rank), but more specialized than females, being exploited by a greater diversity (not necessarily a greater number) of parasite species (given that *d′*, in our case, simultaneously considers the number parasite species and their abundances). This could be the case, for example, for the host-flea networks of *M. rutilus* from Siberia and *A. uralensis* from Slovakia (Table 3). On the one hand, the lesser specialization of female hosts, expressed as nested rank (i.e., concerning pure numbers of parasite species), may be associated with the long time periods they spend in their burrows/nests (especially during their reproductive period when most of the sampling was carried out). These burrows or nests are the main places where nidicolous ectoparasites, such as fleas and mites, reside (Dowling 2006; Krasnov 2008). On the other hand, males are characterized by greater mobility, larger home ranges, and short-term visits to the burrows of other conspecific and heterospecific hosts (Krasnov 2008). This may facilitate their repetitive encounters with various parasites, resulting in high parasite diversity in terms of their relative abundances.

Significant differences between sexes in individual strength were only detected in two host-flea networks and one host-mite network. Strength reflects the dependence of parasites on each host individual (Dormann 2011). Therefore, the females and males of most of the host species considered in our study are equally relevant for all parasite species exploiting this host species. However, the eigenvector centrality values were consistently greater in male than in female hosts (at least, in the networks in which significant differences between sexes were detected). This indicates that males could be more influential than females in these networks, via their contribution to network stability and by sharing parasites mainly with host individuals that, in turn, share many parasites with many other host individuals (Anderson and Sukhdeo 2011; Dallas et al. 2019). The most likely reason behind this is the abovementioned high mobility and large home ranges of males that lead to frequent contacts with other hosts and visits to their burrows.

The extent of the differences between female and male hosts in their network positions was further translated into variation in the entire network structure, in terms of the network specificity, but not in the degree of nestedness, and only for host-flea, but not host-mite, networks. Furthermore, variation in the host-flea network specificity was associated with sex difference variation in the nested rank and species strength, but not in the eigenvector centrality and the degree of specialization. The greater network specificity was detected in networks with lesser levels of female-male differences in their nested ranks and greater levels of differences in their strength. In host-parasite networks, network specificity is positively correlated with the parasite’s level of host-specificity, decreasing competition between them (Blüthgen et al. 2006; de Angeli Dutra and Poulin 2024). Therefore, greater levels of differences between the sexes in their strength are expected to be mainly characteristic for the networks with many host-specific flea species. Network specificity has also been reported to cause a decrease in the degree of nestedness (de Angeli Dutra and Poulin 2024). Consequently, smaller differences between female and male hosts in their nested ranks observed in the highly specific networks suggest that greater differences in nested ranks should be found in highly nested networks. However, we did not find any association between differences in nested ranks and the degree of the network nestedness, although there was a negative correlation between network specificity and the degree of nestedness (Spearman *r* = − 0.81, *p* < 0.05). We recognize that these explanations are highly speculative and warrant further investigations.

An important finding of this study is that differences in the network positions between female and male hosts were detected (a) in some, but not other, host species; (b) in either the host-flea or the host-mite networks (but not both) of the same host species within a region; and (c) in either the host-flea or the host-mite networks of the same host species but only in either Siberia or Slovakia. This variation likely results from multiple differences between hosts and their interactions with fleas or mites in different environments. For example, *Apodemus* mice are mostly solitary (e.g., Stradiotto et al. 2009), whereas many *Microtus* voles are social (e.g., Dobly and Rozenfeld 2000), which might cause a greater frequency of ectoparasite exchange between females and males of the latter than of the former. Sex differences in spatial behavior may or may not occur in the same species in different localities, depending on environmental factors (e.g., Bowers et al. 1996). Sex differences in immunocompetence that may affect the position of a female or a male in a host-parasite network are expressed differently depending on environmental factors and population density (Beldomenico et al. 2008). Environmental factors may also affect the structure of ectoparasite infracommunities (i.e., parasite assemblages on host individuals) (Krasnov et al. 2019). This may lead to differential network structure patterns in the same host-parasite associations but from different localities (Junker et al. 2022). The combined effect of the environment on sex differences in spatial behavior or immunity and ectoparasite infracommunity structure could thus explain why the network positions differ between female and male hosts in some, but not other, environments. In addition, the pattern of parasitism differs substantially between fleas and mites, with fleas being obligatory haematophagous, whereas many gamasid mite species only facultatively feed on host blood (Radovsky 1985). This results in different structural patterns of host-flea and host-mite networks, including (but not limited to; see Krasnov et al. 2019) the effect of individual host sex on its network position.

In conclusion, the answer to the question “do positions in the individual-based host-ectoparasite networks differ between female and male hosts?” is not straightforward. These positions may or may not differ depending on a complex interplay of host-associated, parasite-associated, and environmental factors.

## Supplementary Information

Below is the link to the electronic supplementary material.

Supplementary file1 (DOCX 38 KB)

## Data Availability

The data can be obtained from the corresponding author (B.R.K.) upon request.

## References

[CR1] Alarcon R, Waser NM, Ollerton J (2008) Year-to-year variation in the topology of a plant-pollinator interaction network. Oikos 117:1796–1807. 10.1111/j.0030-1299.2008.16987.x

[CR2] Allesina S, Pascual M (2009) Googling food webs: can an eigenvector measure species’ importance for coextinctions? PLoS Comp Biol 5:e1000494. 10.1371/journal.pcbi.1000494 10.1371/journal.pcbi.1000494 PMC2725316 19730676

[CR3] Almeida-Neto M, Guimaraes P, Guimaraes PR Jr, Loyola RD, Ulrich W (2008) A consistent metric for nestedness analysis in ecological systems: reconciling concept and measurement. Oikos 117:1227–1239. 10.1111/j.0030-1299.2008.16644.x

[CR4] Anderson MJ (2001) A new method for non-parametric multivariate analysis of variance. Austral Ecol 26:32–46. 10.1111/j.1442-9993.2001.01070.pp.x

[CR5] Anderson TK, Sukhdeo MV (2011) Host centrality in food web networks determines parasite diversity. PLoS ONE 6:e26798. 10.1371/journal.pone.0026798 22046360 10.1371/journal.pone.0026798 PMC3201966

[CR6] Bascompte J, Jordano P (2013) Mutualistic networks (monographs in population biology, 53). Princeton Univ Press, Princeton

[CR7] Bascompte J, Jordano P, Melián CJ, Olesen JM (2003) The nested assembly of plant-animal mutualistic networks. Proc Natl Acad Sci USA 100:9383–9387. 10.1073/pnas.1633576100 12881488 10.1073/pnas.1633576100 PMC170927

[CR8] Bascompte J, Jordano P, Olesen JM (2006) Asymetric coevolutionary networks facilitate biodiversity maintenance. Science 312:431–433. 10.1126/science.11234 16627742 10.1126/science.1123412

[CR9] Behnke JM, Lewis JW, Mohd Zain SN, Gilbert FS (1999) Helminth infections in *Apodemus sylvaticus* in southern England: interactive effects of host age, sex and year on the prevalence and abundance of infections. J Helminthol 73:31–44. 10.1017/S0022149X00700162 10431369

[CR10] Beldomenico PM, Telfer S, Gebert S, Lukomski L, Bennett M, Begon M (2008) The dynamics of health in wild field vole populations: a haematological perspective. J Anim Ecol 77:984–997. 10.1111/j.1365-2656.2008.01413.x 18564292 10.1111/j.1365-2656.2008.01413.x PMC2980900

[CR11] Blüthgen N (2010) Why network analysis is often disconnected from community ecology: a critique and an ecologist’s guide. Basic Appl Ecol 11:185–195. 10.1016/j.baae.2010.01.001

[CR12] Blüthgen N, Menzel F, Blüthgen N (2006) Measuring specialization in species interaction networks. BMC Ecol 6:1–12. 10.1186/1472-6785-6-9 16907983 10.1186/1472-6785-6-9 PMC1570337

[CR13] Blüthgen N, Menzel F, Hovestadt T, Fiala B, Blüthgen N (2007) Specialization, constraints, and conflicting interests in mutualistic networks. Curr Biol 17:341–346. 10.1016/j.cub.2006.12.039 17275300 10.1016/j.cub.2006.12.039

[CR14] Bonacich P (1987) Power and centrality: a family of measures. Am J Sociol 92:1170–1182. 10.1086/228631

[CR15] Bowers MA, Gregario K, Brame CJ, Matter SF, Dooley JL Jr (1996) Use of space and habitats by meadow voles at the home range, patch and landscape scales. Oecologia 105:107–115. 10.1007/BF00328798 28307129 10.1007/BF00328798

[CR16] Brooks ME, Kristensen K, van Benthem KJ, Magnusson A, Berg CW, Nielsen A, Skaug HJ, Maechler M, Bolker BM (2017) glmmTMB balances speed and flexibility among packages for zero-inflated generalized linear mixed modeling. The R Journal 9:378–400. 10.32614/RJ-2017-066

[CR17] Christe P, Glaizot O, Evanno G, Bruyndonckx N, Devevey G, Yannic G, Patthey P, Maeder A, Vogel P, Arlettaz R (2007) Host sex and ectoparasites choice: preference for, and greater survival on female hosts. J Anim Ecol 76:703–710. 10.1111/j.1365-2656.2007.01255.x 17584376 10.1111/j.1365-2656.2007.01255.x

[CR18] Collyer ML, Adams DC (2018) RRPP: an R package for fitting linear models to high-dimensional data using residual randomization. Methods Ecol Evol 9:1772–1779. 10.1111/2041-210X.13029

[CR19] Córdoba-Aguilar A, Munguía-Steyer R (2013) The sicker sex: understanding male biases in parasitic infection, resource allocation and fitness. PLoS ONE 8:e76246. 10.1371/journal.pone.0076246 24194830 10.1371/journal.pone.0076246 PMC3806765

[CR20] Cozzarolo CS, Sironi N, Glaizot O, Pigeault R, Christe P (2019) Sex-biased parasitism in vector-borne disease: vector preference? PLoS ONE 14. 10.1371/journal.pone.0216360 10.1371/journal.pone.0216360 PMC6497283 31048933

[CR21] Csardi G, Nepusz T (2006) The igraph software package for complex network research. InterJ Complex Sys 1695. https://igraph.org

[CR22] Csárdi G, Nepusz T, Traag V, Horvát Sz, Zanini F, Noom D, Müller K (2023) igraph: network analysis and visualization in R. 10.5281/zenodo.7682609, R package version 1.5.1, https://CRAN.R-project.org/package=igraph

[CR23] Dallas T, Han BA, Nunn CL, Park AW, Stephens PR, Drake JM (2019) Host traits associated with species roles in parasite sharing networks. Oikos 128:23–32. 10.1111/oik.05602

[CR24] de Angeli DD, Poulin R (2024) Network specificity decreases community stability and competition among avian haemosporidian parasites and their hosts. Global Ecol Biogeogr 33:e13831. 10.1111/geb.13831

[CR25] Delmas E, Besson M, Brice MH, Burkle LA, Dalla Riva GV, Fortin MJ, Gravel D, Guimarães PR Jr, Hembry DH, Newman EA, Olesen JM, Pires MM, Yeakel JD, Poisot T (2019) Analysing ecological networks of species interactions. Biol Rev 94:16–36. 10.1111/brv.12433 29923657 10.1111/brv.12433

[CR26] Dick CW, Gannon MR, Little WE, Patrick MJ (2003) Ectoparasite associations of bats from central Pennsylvania. J Med Entomol 40:813–819. 10.1603/0022-2585-40.6.813 14765658 10.1603/0022-2585-40.6.813

[CR27] Dobly A, Rozenfeld M (2000) Burrowing by common voles *(Microtus arvalis*) in various social environments. Behaviour 137:1443–1462. 10.1163/156853900502664

[CR28] Dormann CF (2011) How to be a specialist? Quantifying specialization in pollination networks. Network Biol 1:1–20

[CR29] Dormann CF, Gruber B, Fruend J (2008) Introducing the bipartite package: analysing ecological networks. R News 8:8–11

[CR30] Dowling APG (2006) Mesostigmatid mites as parasites of small mammals: systematics ecology, and the evolution of parasitic associations. In Morand S Krasnov BR Poulin R (eds) Micromammals and macroparasites. From evolutionary ecology to management. Springer Tokyo. 103–117. 10.1007/978-4-431-36025-4_7

[CR31] Ferrari N, Cattadori I, Nespereira J, Rizzolli AP, Hudson PJ (2004) The role of host sex in parasite dynamics: field experiments on the yellow-necked mouse *Apodemus flavicollis*. Ecol Lett 7:88–94. 10.1046/j.1461-0248.2003.00552.x

[CR32] Folstad I, Karter AJ (1992) Parasites, bright males, and the immunocompetence handicap. Am Nat 139:603–622. 10.1086/285346

[CR33] Freckleton RP, Harvey PH, Pagel M (2002) Phylogenetic analysis and comparative data: a test and review of evidence. Am Nat 160:712–726. 10.1086/343873 18707460 10.1086/343873

[CR34] Garcia LV (2004) Escaping the Bonferroni iron claw in ecological studies. Oikos 105:657–663. 10.1111/j.0030-1299.2004.13046.x

[CR35] Godfray SS (2013) Networks and the ecology of parasite transmission: a framework for wildlife parasitology. Int J Parasitol Parasites Wildl 2:235–245. 10.1016/j.ijppaw.2013.09.001 24533342 10.1016/j.ijppaw.2013.09.001 PMC3862525

[CR36] Grear DA, Perkins SE, Hudson PJ (2009) Does elevated testosterone result in increased exposure and transmission of parasites? Ecol Lett 12:528–537. 10.1111/j.1461-0248.2009.01306.x 19392718 10.1111/j.1461-0248.2009.01306.x

[CR37] Grear DA, Luong LT, Hudson PJ (2012) Sex-biased transmission of a complex life-cycle parasite: why males matter. Oikos 121:1446–1453. 10.1111/j.1600-0706.2012.20358.x

[CR38] Guimerà R, Amaral LN (2005) Functional cartography of complex metabolic networks. Nature 433:895–900. 10.1038/nature03288 15729348 10.1038/nature03288 PMC2175124

[CR39] Hillegass MA, Waterman JM, Roth JD (2008) The influence of sex and sociality on parasite loads in an African ground squirrel. Behav Ecol 19:1006–1011. 10.1093/beheco/arn070

[CR40] Jordán F, Scheuring I (2004) Network ecology: topological constraints on ecosystem dynamics. Phys Life Rev 1:139–172. 10.1016/j.plrev.2004.08.001

[CR41] Jordano P (1987) Patterns of mutualistic interactions in pollination and seed dispersal–connectance, dependence asymmetries and coevolution. Am Nat 129:657–677. 10.1086/284665

[CR42] Junker K, Boomker J, Horak IG, Krasnov BR (2022) Impact of host sex and age on the diversity of endoparasites and structure of individual-based host-parasite networks in nyalas (*Tragelaphus angasii* Angas) from three game reserves in KwaZulu-Natal province, South Africa. Parasitol Res 121:3249–3267. 10.1007/s00436-022-07653-x 36071296 10.1007/s00436-022-07653-x

[CR43] Khokhlova IS, Serobyan V, Degen AA, Krasnov BR (2011) Discrimination of host sex by a haematophagous ectoparasite. Anim Behav 81:275–281. 10.1016/j.anbehav.2010.10.018

[CR44] Kiffner C, Vor T, Hagedorn P, Niedrig M, Ruhe F (2011) Factors affecting patterns of tick parasitism on forest rodents in tick-borne encephalitis risk areas, Germany. Parasitol Res 108:323–335. 10.1007/s00436-010-2065-x 20878183 10.1007/s00436-010-2065-x PMC3024494

[CR45] Krasnov BR (2008). Functional and evolutionary ecology of fleas. a model for ecological parasitology. Cambridge University Press, Cambridge, UK

[CR46] Krasnov BR, Matthee S (2010) Spatial variation in gender-biased parasitism: host-related, parasite-related and environment-related effects. Parasitology 137:1526–1537. 10.1017/S0031182010000454 10.1017/S0031182010000454 20550754

[CR47] Krasnov BR, Morand S, Hawlena H, Khokhlova IS, Shenbrot GI (2005) Sex-biased parasitism, seasonality and sexual size dimorphism in desert rodents. Oecologia 146:209–217. 10.1007/s00442-005-0189-y 16025350 10.1007/s00442-005-0189-y

[CR48] Krasnov BR, Stanko M, Matthee S, Laudisot A, Leirs H, Khokhlova IS, Korallo-Vinarskaya NP, Vinarski MV, Morand S (2011) Male hosts drive infracommunity structure of ectoparasites. Oecologia 166:1099–1010. 10.1007/s00442-011-1950-z 21409449 10.1007/s00442-011-1950-z

[CR49] Krasnov BR, Bordes F, Khokhlova IS, Morand S (2012) Gender-biased parasitism in small mammals: patterns, mechanisms, consequences. Mammalia 76:1–13. 10.1515/mammalia-2011-0108

[CR50] Krasnov BR, Shenbrot GI, Korallo-Vinarskaya NP, Vinarski MV, van der Mescht L, Warburton EM, Khokhlova IS (2019) Do the pattern and strength of species associations in ectoparasite communities conform to biogeographic rules? Parasitol Res 118:1113–1125. 10.1007/s00436-019-06255-4 30778750 10.1007/s00436-019-06255-4

[CR51] Matthee S, McGeoch MA, Krasnov BR (2009) Parasite-specific variation and the extent of male-biased parasitism; an example with a South African rodent and ectoparasitic arthropods. Parasitology 137:651–660. 10.1017/S0031182009991338 19835648 10.1017/S0031182009991338

[CR52] Moore SL, Wilson K (2002) Parasites as a viability cost of sexual selection in natural populations of mammals. Science 297:2015–2018. 10.1126/science.1074196 12242433 10.1126/science.1074196

[CR53] Moran MD (2003) Arguments for rejecting the sequential Bonferroni in ecological studies. Oikos 100:403–405. 10.1034/j.1600-0706.2003.12010.x

[CR54] Morand S, Göuy de Bellocq J, Stanko M, Miklišova D (2004) Is sex-biased ectoparasitism related to sexual size dimorphism in small mammals of Central Europe? Parasitology 129:505–510. 10.1017/s0031182004005840 15521640 10.1017/s0031182004005840

[CR55] Nakagawa S (2004) A farewell to Bonferroni: the problems of low statistical power and publication bias. Behav Ecol 15:1044–1045. 10.1093/beheco/arh107

[CR56] Nielsen A, Bascompte J (2007) Ecological networks, nestedness and sampling effort. J Ecol 95:1134–1141. 10.1111/j.1365-2745.2007.01271.x

[CR57] Opsahl T (2009) Structure and evolution of weighted networks. Dissertation, University of London (Queen Mary College)

[CR58] Perkins SE, Ferrari MF, Hudson PJ (2008) The effects of social structure and sex-biased transmission on macroparasite infection. Parasitology 135:1561–1569. 10.1017/S0031182008000449 18814808 10.1017/S0031182008000449

[CR59] Perneger TV (1998) What’s wrong with Bonferroni adjustments. Br Med J 316:1236–1238. 10.1136/bmj.316.7139.1236 9553006 10.1136/bmj.316.7139.1236 PMC1112991

[CR60] Poulin R (1996a) Helminth growth in vertebrate hosts: does host sex matter? Int J Parasitol 26:1311–1315. 10.1016/S0020-7519(96)00108-7 9024877 10.1016/s0020-7519(96)00108-7

[CR61] Poulin R (1996b) Sexual inequalities in helminth infections: a cost of being a male? Am Nat 147:287–295. 10.1086/285851

[CR62] R Core Team (2024) R: a language and environment for statistical computing. R Foundation for Statistical Computing, Vienna. https://www.R-project.org

[CR63] Revell LJ (2012) phytools: an R package for phylogenetic comparative biology (and other things). Methods Ecol Evol 3:217–223. 10.1111/j.2041-210X.2011.00169.x

[CR64] Rosso AA, Nicholson DJ, Logan ML, Chung AK, Curlis JD, Degon ZM, Knell RJ, Garner TWJ, McMillan WO, Cox CL (2020) Sex-biased parasitism and expression of a sexual signal. Biol J Linn Soc 131:785–800. 10.1093/biolinnean/blaa162

[CR65] Rothman KJ (1990) No adjustments are needed for multiple comparisons. Epidemiology 1:43–46 2081237

[CR66] Schruth DM (2023) mmodely: modeling multivariate origins determinants - evolutionary lineages in ecology. R package version 0.2.5. https://CRAN.R-project.org/package=mmodely

[CR67] Stanko M, Miklisová D, Goüy de Bellocq J, Morand S (2002) Mammal density and patterns of ectoparasite species richness and abundance. Oecologia 131:289–295. 10.1007/s00442-002-0889-5 28547697 10.1007/s00442-002-0889-5

[CR68] Stephenson JF, Kinsella C, Cable J, van Oosterhout C (2016) A further cost for the sicker sex? Evidence for male-biased parasite-induced vulnerability to predation. Ecol Evol 6:2506–2515. 10.1002/ece3.2049 27066240 10.1002/ece3.2049 PMC4797162

[CR69] Stouffer DB, Sales-Pardo M, Sirer MI, Bascompte J (2012) Evolutionary conservation of species’ roles in food webs. Science 335:1489–1492. 10.1126/science.1216556 22442483 10.1126/science.1216556

[CR70] Stradiotto A, Cagnacci F, Delahay R, Tioli S, Luis N, Rizzoli A (2009) Spatial organization of the yellow-necked mouse: effects of density and resource availability. J Mammal 90:704–714. 10.1644/08-MAMM-A-120R1.1

[CR71] Upham NS, Esselstyn JA, Jetz W (2019) Inferring the mammal tree: species-level sets of phylogenies for questions in ecology, evolution, and conservation. PLoS Biol 17:e3000494. 10.1371/journal.pbio.3000494 31800571 10.1371/journal.pbio.3000494 PMC6892540

[CR72] Venables WN, Ripley BD (2002) Modern applied statistics with S, 4th edn. Springer, New York

[CR73] Vicente-Gonzalez L, Vicente-Villardon JL (2021) PERMANOVA: multivariate analysis of variance based on distances and permutations. R package version 0.2.0. https://CRAN.R-project.org/package=PERMANOVA

[CR74] Xu Z, MacIntosh AJJ, Castellano-Navarro A, Macanás-Martínez E, Suzumura T, Duboscq J (2022) Linking parasitism to network centrality and the impact of sampling bias in its interpretation. PeerJ 10:e14305. 10.7717/peerj.14305 36420133 10.7717/peerj.14305 PMC9677876

[CR75] Zuk M (1996) Disease, endocrine-immune interactions, and sexual selection. Ecology 77:1037–1042. 10.2307/2265574

[CR76] Zuk M, McKean KA (1996) Sex differences in parasite infections: patterns and processes. Int J Parasitol 26:1009–1024. 10.1016/S0020-7519(96)80001-4 8982783

