# Supplementary material for: Do positions in individual-based ectoparasite-small mammal networks differ between female and male hosts?
Source: Parasitol Res. 2025 Apr 29;124(4):45. doi: 10.1007/s00436-025-08492-2 (PMC12040995; doi:10.1007/s00436-025-08492-2)
Supplement: Supplementary file 1 — Supplementary file1 (DOCX 38 KB) [file 436_2025_8492_MOESM1_ESM.docx]

**Supplementary Tables**

**Supplementary Table S1**. Numbers of female and male individuals in host species selected for analyses and parasitized by fleas.

| Region | Species | Number of females | Number of males |
| --- | --- | --- | --- |
| Siberia | *Apodemus agrarius* | 134 | 233 |
|  | *Arvicola amphibius* | 102 | 119 |
|  | *Craseomys rufocanus* | 127 | 185 |
|  | *Microtus agrestis* | 97 | 137 |
|  | *Microtus arvalis* | 40 | 71 |
|  | *Microtus gregalis* | 281 | 429 |
|  | *Microtus middendorfii* | 40 | 42 |
|  | *Microtus oeconomus* | 311 | 408 |
|  | *Myodes glareolus* | 222 | 294 |
|  | *Myodes rutilus* | 706 | 1024 |
|  | *Sicista betulina* | 40 | 110 |
|  | *Sorex araneus* | 804 | 1196 |
|  | *Sorex isodon* | 59 | 63 |
|  | *Sorex minutus* | 43 | 55 |
|  | *Sorex tundrensis* | 42 | 60 |
| Slovakia | *Apodemus agrarius* | 446 | 686 |
|  | *Apodemus flavicollis* | 873 | 1135 |
|  | *Apodemus uralensis* | 124 | 210 |
|  | *Microtus arvalis* | 181 | 160 |
|  | *Myodes glareolus* | 517 | 726 |
|  | *Sorex araneus* | 55 | 79 |

**Supplementary Table S2**. Numbers of female and male individuals in host species selected for analyses and parasitized by gamasid mites.

| Region | Species | Number of females | Number of males |
| --- | --- | --- | --- |
| Siberia | *Apodemus agrarius* | 262 | 359 |
|  | *Arvicola amphibius* | 131 | 148 |
|  | *Craseomys rufocanus* | 50 | 82 |
|  | *Microtus agrestis* | 71 | 117 |
|  | *Microtus arvalis* | 40 | 51 |
|  | *Microtus gregalis* | 410 | 568 |
|  | *Microtus middendorfii* | 40 | 41 |
|  | *Microtus oeconomus* | 523 | 576 |
|  | *Myodes glareolus* | 114 | 154 |
|  | *Myodes rutilus* | 430 | 625 |
|  | *Sicista betulina* | 40 | 74 |
|  | *Sorex araneus* | 528 | 715 |
|  | *Sorex isodon* | 40 | 45 |
|  | *Sorex minutus* | 40 | 42 |
|  | *Sorex tundrensis* | 40 | 53 |
| Slovakia | *Apodemus agrarius* | 810 | 1092 |
|  | *Apodemus flavicollis* | 1180 | 2167 |
|  | *Apodemus uralensis* | 194 | 331 |
|  | *Microtus arvalis* | 272 | 249 |
|  | *Myodes glareolus* | 422 | 581 |
|  | *Sorex araneus* | 40 | 52 |

**Supplementary Table S3**. Flea species recorded on host species selected for analyses.

| Region | Species | Flea species |
| --- | --- | --- |
| Siberia | *Apodemus agrarius* | *Amalareus penicilliger, Amphypsylla kuznetzovi, Amphipsylla sibirica, Ceratophyllus indages, Corrodopsylla birulai, Ctenophthalmus assimilis, Ctenophthalmus pisticus, Frontopsylla elata, Hystrichopsylla talpae, Megabothris calcarifer, Megabothris rectangulatus, Megabothris turbidus, Megabothris walkeri, Neopsylla acanthina, Neopsylla mana, Neopsylla pleskei, Palaeopsylla soricis, Peromyscopsylla bidentata, Peromyscopsylla silvatica* |
|  | *Arvicola amphibius* | *A. penicilliger, A. sibirica, C. indages, C. birulai, C. assimilis, F. elata, H. talpae, M. calcarifer, M. rectangulatus, M. turbidus, M. walkeri, N. acanthina, N. mana, N. pleskei, P. soricis, Rhadinopsylla integella* |
|  | *Craseomys rufocanus* | *A. penicilliger, A. kuznetzovi, A. sibirica, Catallagia dacenkoi, Catallagia ioffi, C. indages, C. birulai, C. assimilis, Ctenophthalmus uncinatus, Doratopsylla dasycnema, F. elata, H. talpae, M. calcarifer, M. rectangulatus, M. turbidus, M. walkeri, N. acanthina, N. mana, N. pleskei, P. soricis, P. bidentata, P. silvatica, R. integella* |
|  | *Microtus agrestis* | *A. penicilliger, A. sibirica, C. indages, C. birulai, Ctenophthalmus arvalis, C. assimilis, C. uncinatus,, D. dasucnema, H. talpae, M. calcarifer, M. rectangulatus, M. turbidus, M. walkeri, N. acanthina, N. mana, N. pleskei, P. soricis, P. bidentata, P. silvatica. R. integella* |
|  | *Microtus arvalis* | *A. penicilliger, A. sibirica, C. indages, C. assimilis, Ctenophthalmus breviatus, C. uncinatus, F. elata, H. talpae, M. calcarifer, M. rectangulatus, M. turbidus, N. acanthina, N. mana, P. soricis, Pectinoctenus pavlovskii, P. sylvatica. R. integella* |
|  | *Microtus gregalis* | *A. penicilliger, A. kuznetzovi, A. sibirica, C. indages, C. arvalis, C. assimilis, C. breviatus, F. elata, H. talpae, M. rectangulatus, M. turbidus, M. walkeri, N. acanthina, N. mana, N. pleskei, P. soricis, P. pavlovskii, P. bidentata, P. silvatica, R. integella* |
|  | *Microtus middendorfii* | *A. penicilliger, A. kuznetzovi, A. sibirica, M. rectangulatus, M. walkeriP. bidentata, P. silvatica* |
|  | *Microtus oeconomus* | *A. penicilliger, C. dacenkoi, C. ioffi, C. birulai, C. assimilis, C. breviatus, C. uncinatus, D. dasycnema, F. elata, H. talpae, M. calcarifer, M. rectangulatus, M. turbidus, M. walkeri, N. acanthina, N. pleskei, P. soricis, P. bidentata, P. silvatica, Rhadinopsylla altaica, R. integella* |
|  | *Myodes glareolus* | *A. penicilliger, Amphipsylla rossica, A. sibirica, C. dacenkoi, C. indages, C. birulai, C. assimilis, C. uncinatus, D. dasycnema, F. elata, H. talpae, M. calcarifer, M. rectangulatus, M. turbidus, M. walkeri, N. acanthina, N. mana, N. pleskei, P. soricis, P. bidentata, P. silvatica* |
|  | *Myodes rutilus* | *A. penicilliger, A. kuznetzovi, A. sibirica, C. dacenkoi, C. ioffi, C. indages, C. birulai, C. assimilis, C. breviatus, C. uncinatus, D. dasycnema, F. elata, H. talpae, M. calcarifer, M. rectangulatus, M. turbidus, M. walkeri, N. acanthina, N. mana, N. pleskei, P. soricis, P. pavlovskii, P. bidentata, P. silvatica, R. integella* |
|  | *Sicista betulina* | *A. penicilliger, A. sibirica, C. indages, C. assimilis, H. talpae, M. rectangulatus, M. turbidus, N. acanthina, N. mana, P. soricis* |
|  | *Sorex araneus* | *A. penicilliger, A. kuznetzovi, A. sibirica, C. dacenkoi, C. indages, C. birulai, C. assimilis, C. breviatus, C. uncinatus, D. dasycnema, F. elata, H. talpae, M. rectangulatus, M. turbidus, M. walkeri, N. acanthina, N. mana, N. pleskei, P. soricis, P. bidentata, P. silvatica, R. integella* |
|  | *Sorex isodon* | *A. penicilliger, C. birulai, C. assimilis, H. talpae, M. rectangulatus, P. soricis* |
|  | *Sorex minutus* | *A. penicilliger, C. birulai, C. assimilis, H. talpae, M. rectangulatus, N. acanthina, P. soricis* |
|  | *Sorex tundrensis* | *A. penicilliger, A. sibirica, C. birulai, C. assimilis, F. elata, H. talpae, M. rectangulatus, P. soricis, P. bidentata, P. silvatica* |
| Slovakia | *Apodemus agrarius* | *Amalaraeus arvicolae, Ceratophyllus sciurorum, Ctenophthalmus agyrtes, C. assimilis, Ctenophthalmus bisoctodentatus, Ctenophthalmus solutus, C. uncinatus, D. dasycnema, Hystrichopsylla orientalis, H. talpae, Leptopsylla segnis, M. turbidus, Nosopsyllus fasciatus, Palaeopsylla similis, P. soricis, P. bidentata, P. sylvatica, R. integella* |
|  | *Apodemus flavicollis* | *A. arvicolae, A. penicilliger, Atyphloceras nuperus, C. sciurorum, C. agyrtes, C. assimilis, C. bisoctodentatus, Ctenophthalmus obtusus, C. solutus, C. uncinatus, D. dasycnema, H. orientalis, H. talpae, L. segnis, M. turbidus, N. fasciatus, P. similis, P. soricis, P. bidentata, P. silvatica, R. integella, Rhadinopsylla penthacanta* |
|  | *Apodemus uralensis* | *C. agyrtes, C. assimilis, C. bisoctodentatus, C. solutus, D. dasycnema, H. orientalis, M. turbidus, N. fasciatus, P. soricis* |
|  | *Microtus arvalis* | *A. penicilliger, A. rossica, C. agyrtes, C. assimilis, Ctenophthalmus obtusus, C. solutus, D. dasycnema, H. orientalis, M. turbidus, N. fasciatus, P. soricis, P. bidentata, R. integella, R. penthacantha* |
|  | *Myodes glareolus* | *A. penicilliger, A. rossica, A. nuperus, C. sciurorum, C. agyrtes, C. assimilis, C. bisoctodentatus, C. obtusus, C. solutus, C. uncinatus, D. dasycnema, H. orientalis, H. talpae, L. segnis, M. turbidus, N. fasciatus, P. soricis, P. bidentata, P. silvatica, R. integella,* *Rhadinopsylla isacantha, Rhadinopsylla mesoides, R. penthacantha* |
|  | *Sorex araneus* | *A. penicilliger, C. ayrtes, C. assimilis, C. solutus, D. dasycnema, H. orientalis, M. turbidus, P. similis, P. soricis, P. bidentata, R. penthacantha* |

**Supplementary Table S4**. Gamasid mite species recorded on host species selected for analyses.

| Region | Species | Gamasid mite species |
| --- | --- | --- |
| Siberia | *Apodemus agrarius* | *Androlaelaps casalis, Androlaelaps dogieli, Androlaelaps fahrenholzi, Eulaelaps kolpakovae, Eulaelaps stabularis, Haemogamasus ambulans, Haemogamasus horridus, Haemogamasus ivanovi, Haemogamasus liponissoides, Haemogamasus mandshuricus, Haemogamasus nidi, Haemogamasus nidiformes, Haemogamasus zachvatkini, Hirstionyssus apodemi, Hirstionyssus criceti, Hirstionyssus eusoricis, Hirstionyssus gudauricus, Hirstionyssus isabellinus, Hirstionyssus sciurinus, Hirstionyssus latiscutatus, Hirstionyssus transiliensis, Hyperlaelaps amphibius, Hyperlaelaps arvalis, Hyperlaelaps microti, Laelaps agilis, Laelaps alaskensis, Laelaps algericus, Laelaps clethrionomydis, Laelaps hilaris, Laelaps muris, Laelaps mulispinosus, Laelaps pavlovskyi, Macrocheles matrius, Macrocheles decumani, Macrocheles glaber, Macrocheles nataliae, Macrocheles rotundiscutus, Myonyssus ingricus* |
|  | *Arvicola amphibius* | *A. fahrenholzi, E. stabularis, H. ambulans, H. mandshuricus, H. nidi, H. nidiformes, H. eusoricis, H. isabellinus, H. transiliensis, H. amphibius, H. arvalis, L. clethrionomydis, L. hilaris, L. muris, L. mulispinosus, L. pavlovskyi, M. matrius, M. decumani, M. glaber* |
|  | *Craseomys rufocanus* | *A. casalis, A. fahrenholzi, E. stabularis, H. ambulans, H. liponissoides, H. nidi, H. nidiformes, H. eusoricis, H. isabellinus, L. clethrionomydis, L. hilaris, L. pavlovskyi, M. glaber, M. nataliae, M. ingricus* |
|  | *Microtus agrestis* | *A. casalis, A. fahrenholzi, E. stabularis, H. ambulans, H. mandshuricus, H. nidi, H. nidiformes, H. eusoricis, H. isabellinus, H. arvalis, L. clethrionomydis, L. hilaris, L. muris, L. pavlovskyi, M. glaber, M. rotundiscutus, M. ingricus* |
|  | *Microtus arvalis* | *A. fahrenholzi, E. stabularis, H. ambulans, H. mandshuricus, H. nidi, H. nidiformes, H. eusoricis, H. gudauricus, H. isabellinus, H. latiscutatus, H. arvalis, L. algericus, L. clethrionomydis, L. hilaris, L. muris, L. pavlovskyi, M. glaber, M. nataliae* |
|  | *Microtus gregalis* | *A. casalis, A. fahrenholzi, E. stabularis, H. ambulans, H. ivanovi, H. liponissoides, H. mandshuricus, H. nidi, H. nidiformes, H. zachvatkini, H. apodemi, H. criceti, H. eusoricis, H. gudauricus, H. isabellinus, H. sciurinus, H. latiscutatus, H. transiliensis, H. amphibius, H. arvalis, L. alaskensis, L. clethrionomydis, L. hilaris, L. muris, L. pavlovskyi, M. matrius, M. decumani, M. glaber, M. nataliae, M. rotundiscutus* |
|  | *Microtus middendorfii* | *H. ambulans, H. liponissoides, H. nidi, H. nidiformes, H. isabellinus, H. arvalis, L. alaskensis, L. clethrionomydis, L. muris, L. pavlovskyi* |
|  | *Microtus oeconomus* | *A. casalis, A. dogieli, A. fahrenholzi, E. kolpakovae, E. stabularis, H. ambulans, H. liponissoides, H. mandshuricus, H. nidi, H. nidiformes, H. apodemi, H. criceti, H. eusoricis, H. gudauricus, H. isabellinus, H. amphibius, H. arvalisL. clethrionomydis, L. hilaris, L. muris, L. mulispinosus, L. pavlovskyi, M. glaber, M. nataliae, M. rotundiscutus, M. ingricus* |
|  | *Myodes glareolus* | *A. casalis, A. fahrenholzi, E. stabularis, H. ambulans, H. mandshuricus, H. nidi, H. nidiformes, H. eusoricis, H. isabellinus, H. transiliensis, H. arvalis, L. clethrionomydis, L. hilaris, L. muris, L. pavlovskyi, M. glaber* |
|  | *Myodes rutilus* | *A. casalis, A. fahrenholzi, E. stabularis, H. ambulans, H. liponissoides, H. mandshuricus, H. nidi, H. nidiformes, H. apodemi, H. eusoricis, H. gudauricus, H. isabellinus, H. latiscutatus, H. transiliensis, H. amphibius, H. arvalis, L. clethrionomydis, L. hilaris, L. muris, L. mulispinosus, L. pavlovskyi, M. decumani, M. glaber, M. nataliae, M. ingricus* |
|  | *Sicista betulina* | *A. fahrenholzi, E. stabularis, H. ambulans, H. mandshuricus, H. eusoricis, H. isabellinus, L. clethrionomydis, L. muris, M. glaber, M. rotundiscutus, M. ingricus* |
|  | *Sorex araneus* | *A. casalis, A. fahrenholzi, E. stabularis, H. ambulans, H. liponissoides, H. mandshuricus, H. nidi, H. nidiformes, H. apodemi, H. eusoricis, H. gudauricus, H. isabellinus, H. amphibius, H. arvalis, L. clethrionomydis, L. hilaris, L. muris, L. mulispinosus, L. pavlovskyi, M. matrius, M. decumani, M. glaber, M. nataliae, M. ingricus* |
|  | *Sorex isodon* | *A. fahrenholzi, E. stabularis, H. ambulans, H. mandshuricus, H. eusoricis, H. isabellinus, M. glaber* |
|  | *Sorex minutus* | *A. fahrenholzi, E. stabularis, H. ambulans, H. nidi, H. eusoricis, H. isabellinus, L. clethrionomydis, L. hilaris, L. pavlovskyi* |
|  | *Sorex tundrensis* | *A. fahrenholzi, E. stabularis, H. ambulans, H. liponissoides, H. mandshuricus, H. nidi, H. apodemi, H. eusoricis, H. isabellinus, H. latiscutatus, H. transiliensis, H. arvalis, L. clethrionomydis, L. mulispinosus, L. pavlovskyi, M. ingricus* |
| Slovakia | *Apodemus agrarius* | *A. casalis, A. fahrenholzi, E. stabularis, Haemogamasus hirsutus, Haemogamasus hirsutosimilis, H. horridus, H. nidi, H. apodemi, H. isabellinus, H. microti, L. agilis, L. clethrionomydis, L. hilaris, Laelaps jettmari, Myonyssus rossicus* |
|  | *Apodemus flavicollis* | *A. casalis, A. fahrenholzi, E. stabularis, H. hirsutus, H. hirsutosimilis, H. horridus, H. nidi, H. apodemi, Hirstionyssus carnifex, H. isabellinus, L. agilis, L. clethrionomydis, L. hilaris, L. jettmari, M. rossicus* |
|  | *Apodemus uralensis* | *A. casalis, A. fahrenholzi, E. stabularis, H. hirsutus, H. nidi, H. apodemi, H. carnifex, H. isabellinus, H. microti, L. agilis, L. clethrionomydis, L. hilaris, L. jettmari, M. rossicus* |
|  | *Microtus arvalis* | *A. fahrenholzi, E. stabularis, H. hirsutus, H. horridus, H. nidi, H. apodemi, H. eusoricis, H. carnifex, H. isabellinus, H. microti, L. agilis, L. hilaris, L. jettmari* |
|  | *Myodes glareolus* | *A. casalis, A. fahrenholzi, E. stabularis, H. hirsutus, H. hirsutosimilis, H. horridus, H. nidi, H. apodemi, H. isabellinus, H. microti, L. agilis, L. clethrionomydis, L. hilaris, L. jettmari, M. rossicus* |
|  | *Sorex araneus* | *A. fahrenholzi, E. stabularis, H. hirsutus, H. nidi, H. apodemi, H. eusoricis, H. microti, L. agilis, L. clethrionomydis, L. hilaris, L. jettmari* |

**Supplementary Table S5**. Median (IQR) values for the numbers of flea individuals recorded on female and male hosts from the two regions.

| Region | Species | Females | Males |
| --- | --- | --- | --- |
| Siberia | *A. agrarius* | 2 (2) | 2 (2) |
|  | *A. amphibius* | 7 (11) | 5.5 (9.25) |
|  | *C. rufocanus* | 2 (2) | 2.5 (3.25) |
|  | *M. agrestis* | 2 (2) | 3 (4.75) |
|  | *M. arvalis* | 2 (3) | 2 (3) |
|  | *M. gregalis* | 2 (1) | 3 (5) |
|  | *M. middendorffii* | 2 (3) | 1 (0.25) |
|  | *M. oeconomus* | 2 (2) | 3 (4) |
|  | *M. glareolus* | 2 (2) | 2 (3) |
|  | *M. rutilus* | 2 (2) | 2 (2) |
|  | *S. betulina* | 1 (1) | 1 (1) |
|  | *S. araneus* | 3 (4) | 1 (1) |
|  | *S. isodon* | 1 (2) | 2 (5) |
|  | *S. minutus* | 2 (2.5) | 1 (1.5) |
|  | *S. tundrensis* | 2 (2) | 2 (3) |
| Slovakia | *A. agrarius* | 1 (2) | 2 (2) |
|  | *A. flavicollis* | 1 (2) | 2 (2) |
|  | *A. uralensis* | 1 (1.75) | 2 (2) |
|  | *M. arvalis* | 2 (3) | 2 (4) |
|  | *M. glareolus* | 2 (4) | 3 (4) |
|  | *S. araneus* | 1 (2) | 2 (3.5) |

**Supplementary Table S6**. Median (IQR) values for the numbers of flea species recorded on female and male hosts from the two regions.

| Region | Species | Females | Males |
| --- | --- | --- | --- |
| Siberia | *A. agrarius* | 1 (1) | 1 (1) |
|  | *A. amphibius* | 2 (2) | 2 (2) |
|  | *C. rufocanus* | 1 (1) | 2 (2) |
|  | *M. agrestis* | 2 (1) | 2 (1.75) |
|  | *M. arvalis* | 1 (1) | 2 (1) |
|  | *M. gregalis* | 1 (1) | 2 (1) |
|  | *M. middendorffii* | 2 (2) | 1 (0) |
|  | *M. oeconomus* | 1 (1) | 2 (2) |
|  | *M. glareolus* | 1 (1) | 1 (1) |
|  | *M. rutilus* | 1 (1) | 1 (1) |
|  | *S. betulina* | 1 (0) | 1 (0.75) |
|  | *S. araneus* | 1 (1) | 1 (0) |
|  | *S. isodon* | 1 (0) | 1 (0) |
|  | *S. minutus* | 1 (0.5) | 1 (0) |
|  | *S. tundrensis* | 1 (1) | 1 (0.25) |
| Slovakia | *A. agrarius* | 1 (1) | 1 (1) |
|  | *A. flavicollis* | 1 (1) | 1 (1) |
|  | *A. uralensis* | 1 (1) | 1 (1) |
|  | *M. arvalis* | 1 (1) | 1 (1) |
|  | *M. glareolus* | 2 (1) | 2 (1) |
|  | *S. araneus* | 1 (1) | 1 (1) |

**Supplementary Table S7**. Median (IQR) values for the numbers of gamasid mite individuals recorded on female and male hosts from the two regions.

| Region | Species | Females | Males |
| --- | --- | --- | --- |
| Siberia | *A. agrarius* | 3 (3) | 2 (3) |
|  | *A. amphibius* | 3.5 (7) | 5 (13) |
|  | *C. rufocanus* | 1 (1) | 1 (1) |
|  | *M. agrestis* | 2 (2) | 2 (2) |
|  | *M. arvalis* | 2.5 (2.75) | 2 (3) |
|  | *M. gregalis* | 3 (6) | 5 (12) |
|  | *M. middendorffii* | 4 (6.25) | 6 (7) |
|  | *M. oeconomus* | 2 (2) | 2 (2) |
|  | *M. glareolus* | 1 (1) | 1 (1) |
|  | *M. rutilus* | 1 (1) | 1 (1) |
|  | *S. betulina* | 1 (0.5) | 1 (1) |
|  | *S. araneus* | 1 (2) | 2 (2) |
|  | *S. isodon* | 1 (0) | 1.5 (1.75) |
|  | *S. minutus* | 1 (1) | 1 (1.25) |
|  | *S. tundrensis* | 1 (1.25) | 1 (1) |
| Slovakia | *A. agrarius* | 2 (3) | 2 (2) |
|  | *A. flavicollis* | 5 (10) | 2 (2) |
|  | *A. uralensis* | 2 (2) | 2 (2) |
|  | *M. arvalis* | 6 (10) | 2 (4) |
|  | *M. glareolus* | 1 (1) | 3 (4) |
|  | *S. araneus* | 1 (0.5) | 2 (3.5) |

**Supplementary Table S8**. Median (IQR) values for the numbers of gamasid mite species recorded on female and male hosts from the two regions.

| Region | Species | Females | Males |
| --- | --- | --- | --- |
| Siberia | *A. agrarius* | 1 (1) | 1 (1) |
|  | *A. amphibius* | 2 (2) | 2 (2) |
|  | *C. rufocanus* | 1 (0) | 1 (0) |
|  | *M. agrestis* | 1 (1) | 1 (1) |
|  | *M. arvalis* | 1 (1) | 1 (1) |
|  | *M. gregalis* | 1 (1) | 2 (2) |
|  | *M. middendorffii* | 2 (2) | 2 (2) |
|  | *M. oeconomus* | 1 (1) | 1 (1) |
|  | *M. glareolus* | 1 (0) | 1 (1) |
|  | *M. rutilus* | 1 (0) | 1 (1) |
|  | *S. betulina* | 1 (0) | 1 (0) |
|  | *S. araneus* | 1 (0) | 1 (1) |
|  | *S. isodon* | 1 (0) | 1 (0) |
|  | *S. minutus* | 1 (0) | 1 (0.25) |
|  | *S. tundrensis* | 1 (0) | 1 (0) |
| Slovakia | *A. agrarius* | 1 (1) | 2 (3) |
|  | *A. flavicollis* | 1 (1) | 7 (9) |
|  | *A. uralensis* | 1 (1) | 2 (3) |
|  | *M. arvalis* | 2 (2) | 6 (13) |
|  | *M. glareolus* | 1 (0) | 2 (4) |
|  | *S. araneus* | 1 (0) | 1 (0) |
